# Epidermoid Cyst of the Cecum Treated by Laparoscopic Colectomy: A Case Report With Histopathology and Literature Review

**DOI:** 10.1155/crgm/6326844

**Published:** 2025-06-13

**Authors:** Ada Firrincieli, Eleonora Nardi, Lavinia Pugliese, Chiara Marconcini, Giovanni Alemanno, Luca Messerini

**Affiliations:** ^1^Section of Anatomic Pathology, Department of Health Sciences, Careggi University Hospital, Florence, Italy; ^2^Histopathology and Molecular Diagnostics, Careggi University Hospital, Florence, Italy; ^3^Radiodiagnostic Unit, Department of Experimental and Clinical Biomedical, Careggi University Hospital, Florence, Italy; ^4^General Emergency and Minimally Invasive Surgery Unit, Careggi University Hospital, Florence, Italy; ^5^Diagnostic and Molecular Pathology, Department of Experimental and Clinical Medicine, Careggi University Hospital, Florence, Italy

**Keywords:** cecum, epidermoid cysts (ECs), histopathologic examination, laparoscopic surgery

## Abstract

**Introduction:** Cecal epidermoid cyst (CEC) is a rare and benign lesion; the origin can be acquired or congenital, but the pathogenesis remains unclear. We present a case report of a patient with a cecal cyst treated by hemicolectomy. Histopathology revealed an epidermoid cyst (EC) of the cecum.

**Case Presentation:** A 28-year-old woman was admitted to the hospital with abdominal pain, without significant past medical history. CT and MRI scans were performed, and a large cystic mass in the anterior portion of the pelvic region was detected. Imaging techniques managed to localize the site and dimensions of the neoplasm; however, they did not provide a conclusive diagnosis. The differential diagnosis was made with appendiceal mucocele, duplication cyst, or endometriotic cyst formation. Laparoscopic right hemicolectomy was performed; the mass did not present with any adhesions with the surrounding organs. Macroscopically, the mass appears as irregular extraluminal cystic lesion arising from the cecal wall of 104 × 83 × 68 mm. Microscopically, the cystic wall was lined by keratinized stratified squamous epithelium. No malignant findings were identified. Thus, the histopathologic evaluation leads to the final diagnosis of EC.

**Conclusions:** ECs are rare benign neoplasms that can be acquired or congenital. They can vary both in their clinical and imaging presentation; the lesion can be associated with nonspecific symptoms or be asymptomatic. A wide heterogeneity both in sex distribution and age is observed. Imaging techniques are useful, but the final diagnosis can be made only after the complete surgical excision of the neoplasm and its histopathological examination.

## 1. Introduction

Epidermoid cysts (ECs) are neoplasms that can develop in different parts of the body such as liver, spleen, kidney, and testis [1]. Those arising from the cecum are extremely rare; only 12 cases have been reported [2]. The origin can be acquired or congenital. Congenital EC is described in patients who do not have previous history of intra-abdominal surgery, abdominal trauma, or chronic inflammation; probably, it is the result of an aberrant ectodermal implantation during embryogenesis [3]. Conversely, the acquired form is associated with a history of abdominal surgery.

Basically, ECs are regarded as having benign characteristics, but a complete tumor resection is required due to the possibility of tumor recurrence or malignant transformation [4, 5].

Laparoscopic surgery has become the standard approach for colorectal surgery, offering shorter incisions, improved cosmesis, and better postoperative outcomes compared with an open approach [6].

We report a case of a 28-year-old woman, with no history of abdominal surgery or trauma, who was diagnosed with a cystic neoplasm of the cecum considered to meet the indications for laparoscopic surgery.

## 2. Case Presentation

A 28-year-old woman was admitted to the hospital with abdominal pain. She did not present prior history of chronic disease or previous surgeries. Vital signs were stable, no fever, vomiting or nausea were present. Physical examination revealed no tenderness in the abdomen, muscle guarding, or rebound tenderness. Laboratory data showed no inflammation or abnormal values.

**Figure 1:**
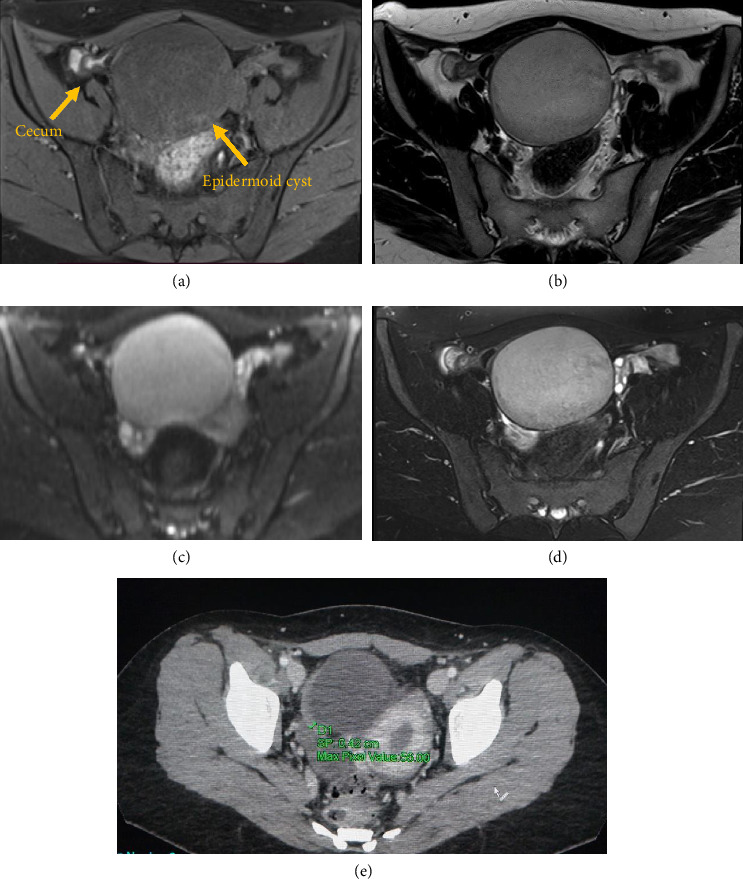
Abdominal MRI. MRI reveals a cystic mass attached to the cecum, appearing hypointense on T1-weighted imaging (a) and hyperintense on T2-weighted imaging (b–d). (a) Axial MRI image T1 TSE Dixon water only of the epidermoid cyst. (b) Axial MRI image T2 TSE, with the fluid and internal debrides of epidermoid cyst. (c) Axial MRI image DWI, b 800, shows the high restriction. (d) Axial MRI image, contrast-enhanced T2 TSE, demonstrates the lack of enhancement. (e) CECT abdomen reveals the appendix of normal dimension (4 mm).

Two abdominal magnetic resonance imaging (MRI) scans were performed, detecting a cystic mass of 104 × 83 × 68 mm in the anterior portion of the pelvic region. This neoplasm was close to the cecum without involving other abdominal organs such as uterus, bladder, sigma, and ovaries. The MRI scan showed a mass with hypoisointense signals on T1-weighted (T1W) images and hyperintense signal on T2-weighted (T2W) TSE images. Irregular heterogeneous areas of low signal intensity were noted in on both T1W and T2W images. Contrast-enhanced T1W images did not demonstrate enhancement. DWI showed high signal intensities (Figure 1). These MRI features can be observed both in ECs and in appendiceal mucocele [7]. However, the radiological assessment was initially misdiagnosed. The differential diagnosis included either a duplication cyst or an endometriotic cyst formation.

**Figure 2:**
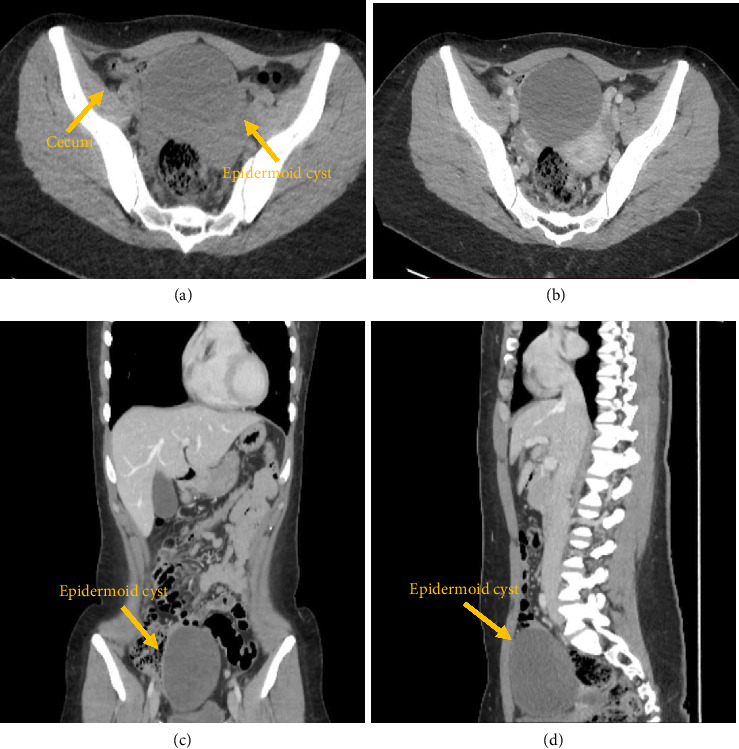
Contrast-enhanced CT. Computed tomography scan image showing a well circumscribed cystic mass adherent to the cecum and containing heterogeneous content of increased attenuation (a–d). (a) Axial CT image before contrast. (b) Axial CT contrast-enhanced image. The wall shows enhancement. (c) Coronal CT contrast-enhanced image shows the craniocaudal extension of the mass. (d) Sagittal CT contrast-enhanced image shows the anteroposterior extension of the mass.

After 1 month, due to the worsening of the symptoms, an abdominal computed tomography (CT) scan was performed, confirming the presence of the cyst in close relation to cecum and ileum. This imaging investigation excluded appendicular mucocele as the appendix was normal in dimension and site (Figure 2).

Both CT and MRI are essential in ECs determinate the correct diagnosis and deciding on the surgical excision strategy [8]. CT scan showed an ovoid mass with fluid density (low attenuation), noninfiltrating, and well encapsulated with a sclerotic wall. The wall enhanced on contrast-enhanced images in the venous phase.

The case was discussed with a multidisciplinary equipped according to the indication for surgery. The procedure was executed with an explorative laparoscopy and the presence of a large neoplasm in the pelvic region was confirmed. This formation was attached to the cecum and the terminal ileum, without involving uterus and adnexa. Due to the nonspecific nature and the presence of numerous lymphadenopathies, a laparoscopic right hemicolectomy with an ileocolic stapled anastomosis was performed. The postoperative course was uneventful, and the patient was discharged on postoperative day 5.

### 2.1. Histopathological Diagnosis

The specimen was sent to the histopathology laboratory to be evaluated both macroscopically and microscopically.

**Figure 3:**
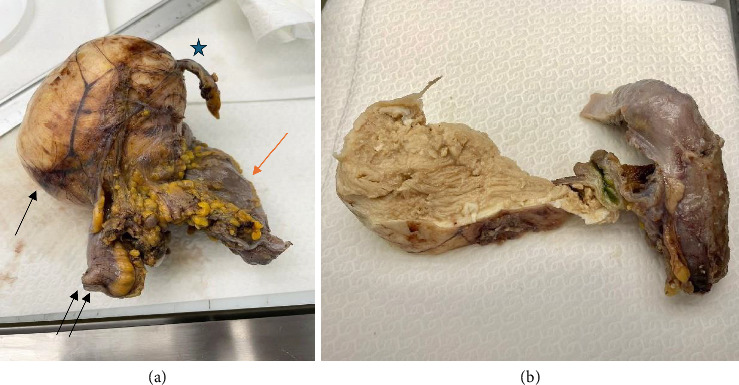
Macroscopic examination. The round mass arising from the cecal wall. The surface of the mass revealed an irregular cystic lesion filled with a yellowish-gray, cheesy material (a-b). In Figure (a), single black arrow shows the cystic mass, single red arrow shows the cecum, double black arrow shows the ileum, and black star shows the appendix.

The main macroscopic findings showed a 10 × 7 × 5 cm, whitish, round mass arising from the cecal wall. There was no apparent communication between the cyst and the mucosa of the cecum that appeared to be intact (Figure 3).

**Figure 4:**
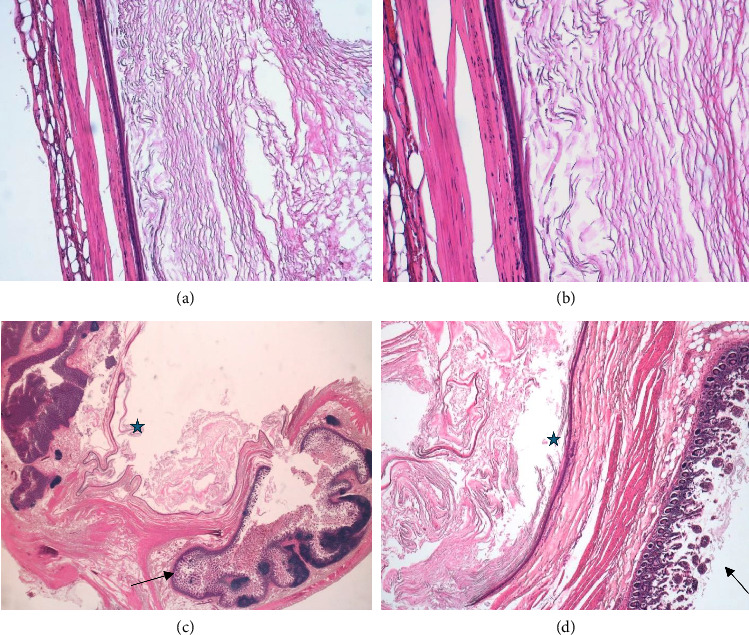
Microscopic examination. The cyst wall (black star) is composed of benign, keratinizing, stratified squamous epithelium with well-formed granular layer and adjacent abundant keratin. H&E × 10 (a) and H&E × 20 (b). The figures (c-d) show the origin of the cyst from the cecum wall (single black arrow); the wall of the cyst is surrounded in its entirety by the intestinal muscularis propria.

Microscopic examination revealed that the wall of the cyst was surrounded in its entirety by a cecal muscularis propria. The cyst wall was exclusively lined with a mature keratinized and stratified squamous epithelium with a granular layer, and focal area of cystic wall showed nonkeratinizing squamous epithelium without a granular layer. The lumen of the cyst was filled with mature, dense keratin (Figure 4).

The final histopathological diagnosis was the EC of the cecum.

## 3. Discussion

ECs of the cecum are extremely rare; however, similar cysts of internal organs have been reported involving the testis, epididymis, spleen, accessory spleen, kidney, and liver [1]. These cysts are generally accepted to be sequestration cysts with congenital or acquired origin.

Congenital ECs are related to inclusion of ectodermal elements at time of closure of neural groove or when epithelial surfaces fuse. Andiran et al. [9] suggested that the inclusion or closure line of the epidermal structure may occur when the cecum re-enters the abdominal cavity during intrauterine rotation in the final steps of gut development. This encasing of an epidermal structure may result in later development of a cecal epidermoid cyst (CEC). Acquired ECs are attributed to the iatrogenic implantation of epidermal fragments through surgical devices in previous abdominal operations, such as appendectomy or caesarean section [10, 11] or due to trauma.

Very few cases of EC of the cecum have been reported (Table 1).

Park et al. reported 9 cases including 3 cases (33.3%) of which are of acquired variety as have been associated with a history of abdominal surgery. Two had an appendectomy 12 years and 16 years before the diagnosis of a CEC [10, 11]. Furthermore, the case reported in 2012 was of a 31-year-old woman, with a previous caesarean surgery, who got admitted for an adnexal mass [14]. The other cases feature patients with no history of abdominal surgery, trauma, or chronic inflammation, and their CECs were considered as congenital lesions. Most of the reported cases in literature present patients with a history of chronic abdominal pain and other unspecific symptoms (nausea, asthenia, abdominal swelling, etc.) with the development of a palpable mass in the right lower abdominal quadrant. In some patients, ECs of the cecum are completely asymptomatic, and they are occasionally found during radiological exams performed for other reasons.

In our case, the patient had abdominal pain and MRI scans were performed detecting a cystic mass in the anterior portion of the pelvic region and after 1 month, and due to the worsening of the symptoms, an abdominal CT scan was performed, confirming the presence of the cyst. Physical examination and radiological findings are unspecific; in fact, none of the previous reports described a precise preoperative diagnosis. On CT, EC appears as a well-demarcated, low-density mass with enhancement of the capsule following contrast administration [18]. On MRI scan, the tumor appears hypointense on T1W imaging and hyperintense on T2W imaging.

The differential diagnosis is seldom taken into consideration in patients with cecal cystic lesions because ECs of the cecum are mainly located in the subserosal area [1, 3, 9, 12–14], as in our case. These cysts may be confused with other intra-abdominal cystic lesions, including appendiceal mucocele, duplication or mesenteric cyst, lymphatic cysts, gastrointestinal stromal tumors, and, in female patients, a right adnexal mass or cyst [2] or endometriotic cyst. Joo- Young Na et al. reported a case of congenital CEC in neonate initially misdiagnosed with retroperitoneal teratoma [17]. In the present case, the cystic mass showed on CT scan was suspected to be either an appendicular mucocele, a duplication cyst, or endometriotic cyst. Complete surgical excision of ECs is recommended to prevent recurrence and potential malignant transformation [19]. The residual tissue and cyst lining may cause recurrence; its rate has been reported as 2% [5]. In the present case, the laparoscopic right hemicolectomy with an ileocolic-stapled anastomosis was performed and the neoplasm was completely resected, as in cases of malignancy. Although there is no consensus in the literature regarding follow-up protocols after surgical resection of CECs, most authors suggest a long-term clinical and radiological surveillance, particularly in cases with uncertain margins or suspicion of malignant transformation.

In the literature, the laparoscopic approach was described as beneficial for both diagnosis and treatment of ECs. Only the most recent cases were treated by laparoscopic surgery, as in our case. This minimally invasive surgery procedure has recently become a popular approach to colon disease due to better short-term outcomes including reduced blood loss, improved intestinal function, and shorter duration of hospitalization [6]. Despite uncertainty about the origin of the mass, after the complete surgical excision has been performed, the histological examination of the specimen is the only investigation that provides a final diagnosis.

Macroscopically, these masses appear as solid neoplasms of variable size, up to 10 cm, as in our case. Microscopically, the cyst wall is lined by benign, keratinizing, stratified squamous epithelium with well-formed granular layer and abundant keratin but without skin specialized structures. The absence of skin specialized structures differentiates EC from dermoid cyst. There is no communication between the unilocular cyst and the normal colic mucosa.

## 4. Conclusions

ECs are rare and generally benign neoplasms. Several cases have been reported, showing a wide range of heterogeneity in both sex distribution and age.

Considering our case and literature review, the clinical presentation and the instrumental investigations can vary, making an immediate diagnosis difficult.

Therefore, the possibility of an EC should be taken into account in the differential diagnosis of submucosal or intramuscular masses in the cecal region.

## Figures and Tables

**Table 1:** Reported cases of cecal epidermoid cysts.

#	Age	Sex	Symptoms	Initial diagnogis	Operation history	Location	Dimensions (cm)	Year	Reference
1	53	M		Right lower abdominal mass caecal defect	Appendicectomy	Intramural		1961	[10]
2	27	F		Right ovarian cyst ovarian torsion chronic appendicitis	None	Subserosal		1965	[12]
3	71	M		Intramural cecal mass	Appendicectomy	Intramural		1969	[11]
4	8	F	Periumbilical abdominal pain for 2 days	Right lower abdominal cyst	None	Subserosal	3 × 3 × 2	1999	[9]
5	67	M	Intermittent nausea vomiting abdominal pain (3 months)	Duplication cyst	None	Subserosal	5 × 6	2002	[13]
6	75	M		Appendiceal mucocele	None	Subserosal		2006	[1]
7	31	F		Adnexal mass	Cesarean surgery	Subserosal		2012	[14]
8	54	M	Intermittent right iliac fossa pain (3 months)	Mesenteric cyst	None	Subserosal	7 × 8 × 6	2013	[3]
9	63	F	No symptoms (incidental finding)	Gastrointestinal stromal tumor (GIST)	Submandibular gland excision due to Wharton's duct stone	Intramural	3.8 (diameter)	2015	[2]
10	20	F	Abdominal pain in the right lower quadrant	Gastrointestinal stromal tumor (GIST) OR Duplication cyst	None		5.6 × 4.5	2021	[15]
11	51	M	No symptoms (incidental finding)	Appendiceal mucocele	None		7.5 (diameter)	2021	[16]
12	38 weeks	M	No symptoms (incidental finding)	Retroperitoneal teratoma	None	Subserosal	5.0 × 6.6	2021	[17]
13	24	F	Abdominal pain in the right lower quadrant	Appendicular mucocele, duplication cyst, or endometriotic formation	None	Subserosal	10.4 × 8.3	Our case	

## Data Availability

The data used to support the findings of this study are included within the article.

## Nomenclature

ECs: Epidermoid cysts
CEC: Cecal epidermoid cyst
MRI: Magnetic resonance imaging
CT: Computed tomography
T1W: T1-weighted images
T2W: T2-weighted images
DWI: Diffusion-weighted imaging
H&E: Hematoxylin and eosin
